# Molecular Dynamics of DHHC20 Acyltransferase Suggests Principles of Lipid and Protein Substrate Selectivity

**DOI:** 10.3390/ijms23095091

**Published:** 2022-05-03

**Authors:** Irina Panina, Nikolay Krylov, Mohamed Rasheed Gadalla, Elena Aliper, Larisa Kordyukova, Michael Veit, Anton Chugunov, Roman Efremov

**Affiliations:** 1 Shemyakin-Ovchinnikov Institute of Bioorganic Chemistry, Russian Academy of Sciences, 117997 Moscow, Russia; irinaspanina@gmail.com (I.P.); krylovna@gmail.com (N.K.); la.marelle@gmail.com (E.A.); 2 International Laboratory for Supercomputer Atomistic Modelling and Multi-Scale Analysis, National Research University Higher School of Economics, 101000 Moscow, Russia; 3 Department of Veterinary Medicine, Institute of Virology, Free University Berlin, 14163 Berlin, Germany; m.rasheed@fu-berlin.de (M.R.G.); michael.veit@fu-berlin.de (M.V.); 4 Department of Virology, Faculty of Veterinary Medicine, Cairo University, Giza 12211, Egypt; 5 Belozersky Institute of Physico-Chemical Biology, Lomonosov Moscow State University, 119899 Moscow, Russia; kord@belozersky.msu.ru; 6 Moscow Institute of Physics and Technology (State University), Dolgoprudny, 141701 Moscow, Russia

**Keywords:** DHHC-PATs, S-acylation, palmitoylation, acyl CoA, molecular dynamics

## Abstract

Lipid modification of viral proteins with fatty acids of different lengths (S-acylation) is crucial for virus pathogenesis. The reaction is catalyzed by members of the DHHC family and proceeds in two steps: the autoacylation is followed by the acyl chain transfer onto protein substrates. The crystal structure of human DHHC20 (hDHHC20), an enzyme involved in the acylation of S-protein of SARS-CoV-2, revealed that the acyl chain may be inserted into a hydrophobic cavity formed by four transmembrane (TM) α-helices. To test this model, we used molecular dynamics of membrane-embedded hDHHC20 and its mutants either in the absence or presence of various acyl-CoAs. We found that among a range of acyl chain lengths probed only C16 adopts a conformation suitable for hDHHC20 autoacylation. This specificity is altered if the small or bulky residues at the cavity’s ceiling are exchanged, e.g., the V185G mutant obtains strong preferences for binding C18. Surprisingly, an unusual hydrophilic ridge was found in TM helix 4 of hDHHC20, and the responsive hydrophilic patch supposedly involved in association was found in the 3D model of the S-protein TM-domain trimer. Finally, the exchange of critical Thr and Ser residues in the spike led to a significant decrease in its S-acylation. Our data allow further development of peptide/lipid-based inhibitors of hDHHC20 that might impede replication of Corona- and other enveloped viruses.

## 1. Introduction

S-acylation is the post-translational attachment of fatty acids to cysteine residues via thioester bonds. Thousands of integral and soluble proteins are targets of this most common lipid modification of proteins [1]. It is catalyzed by a family of polytopic integral membrane enzymes known as DHHC protein acyltransferases (DHHC-PATs), or ZDHHC-proteins. These enzymes have a conserved Asp-His-His-Cys (DHHC) sequence, a variant of the zinc finger motif, located within a ~50 amino acid long cysteine-rich domain (CRD) [2,3]. To date, 23 human DHHC-PATs are known, which show distinct, but overlapping substrate specificities [4]. Most of them are abundant in many tissues, but some are only expressed in a limited number of cell types. Whilst the majority of DHHC-PATs are localized in Golgi membranes, a few are found in the endoplasmic reticulum (ER) or the plasma membrane [5,6]. There has been an increasing amount of evidence confirming the importance of S-acylation in human physiology and the course of pathological processes [7,8]. The activity of ZDHHCs within a cell is counteracted by acyl-protein thioesterases [9].

DHHCs exhibit a two-step reaction mechanism [10,11,12]. First, at the autoacylation stage, an acyl-CoA (coenzyme A) is used to generate an acyl-enzyme intermediate, after which the acyl group is transferred from this intermediate to the thiol group of a Cys residue within the substrate protein. DHHC-PATs thus have two substrates: the lipid (acyl-CoA) and the protein to be acylated.

Although palmitate (C16:0) is predominant in most S-acylated proteins, other types of fatty acids could be also attached, e.g., stearate (C18:0), oleate (C18:1), or palmitoleate (C16:1) [8,13,14,15]. The significance of stearate and/or oleate binding for switching metabolic pathways was shown for some regulatory cellular proteins, such as human transferrin receptor 1 (TFR1) [15] and Guanine nucleotide-binding protein G(I) alpha-1 subunit (GNAI) [8]. However, the role of the lipid environment, which is different between cellular organelles [16] in the functioning of any of the members of the DHHC-PAT family and/or for the attachment of certain types of fatty acids to specific proteins, is absolutely unknown.

Using MALDI-TOF mass spectrometry analysis we had earlier found that the location of an acylation site relative to the TM span of influenza virus hemagglutinin (HA) and other integral viral glycoproteins responsible for membrane fusion can act as the main signal for stearate (C18:0) attachment, while cytoplasmic cysteines only bind palmitates [17,18,19]. These results suggest that viral glycoproteins are acylated by two different enzymes, one with a high specificity for Pal-CoA that acylates cytoplasmic cysteines and the other with a preference for Stear-CoA that recognizes cysteines at the membrane–cytosol interface. The functional consequences of differential fatty acylation have been proposed for several biological systems [8,13,14,15], but the molecular basis of fatty acid selectivity of DHHC-enzymes is not yet understood [20]. In general, one can hypothesize that at least three different factors must be taken into consideration, and for each of them some confirming data have been obtained: (1) the specificity of the enzyme [10,20]; (2) the abundance of the acyl-CoA in the cell and/or in the membrane where acylation occurs [8]; and (3) certain structural signals within the protein substrate [17,18,19,21].

Only some DHHC-PATs have been characterized with respect to their lipid substrate specificity. Using purified ZDHHCs and substrate proteins it was demonstrated that ZDHHC3 has a strong preference for Pal-CoA over Stear-CoA, whereas ZDHHC2 displays no clear preference [10]. Using labeling of cells with various fatty acid analogues and subsequent click-chemistry-based detection, it was reported that ZDHHC3 clearly prefers Myr-CoA (C14:0) and Pal-CoA over Stear-CoA, while the highly related ZDHHC7 enzyme prefers C18:0 [20]. Furthermore, ZDHHC17 prefers C16:0/C18:0 over C14:0; ZDHHC5, ZDHHC11, and ZDHHC15 prefer C14:0/C16:0; ZDHHC2 and ZDHHC4 display no clear fatty acid preference; and ZDHHC23 exhibits a strong preference for C18:0 [20]. In an autoacylation assay, ZDHHC20 prefers palmitate to myristate and stearate [11].

So far, DHHC20 is one of the best functionally characterized acyltransferases in their family. Several protein substrates have been experimentally confirmed for DHHC20, including those involved in cellular transformation and cancer, e.g., the epidermal growth factor receptor (EGFR) [22,23], as well as many proteins of enveloped viruses [24]. It is noteworthy that dependent on DHHC20 is the S-acylation of viral fusion glycoproteins, including those from such important pathogens as the influenza virus [25] and SARS-CoV-2 [26]. Gadalla et al. identified DHHC2, 8, 15, and 20 as essential to the S-acylation of HA, a major virus antigen, and, by consequence, to virus infectivity [27]. Puthenveetil et al. proposed DHHCs 2, 3, 6, 11, 20, 21, and 24 as putative S-acylation enzymes for the SARS-CoV-2 spike protein (Spike) [28], while in other studies data were obtained indicating that Spike acylation is effectuated via the sequential action of DHHC20 and 9 [26]. The M2 protein of Influenza A virus and E protein of SARS-CoV-2, both operating as ion channels, also engage DHHC20 among a number of enzymes [26,27].

Recently, the first atomic structures of two DHHC-PATs—human DHHC20 (hDHHC20) and zebrafish DHHC15—were determined using X-ray crystallography [11]. The 3D structures include four transmembrane (TM) helices forming a teepee-like structure with an inner cavity accommodating a fatty acid entity and a DHHC-motif located at the membrane–cytosol interface. The active site has a catalytic-triad-like arrangement of aspartic acid and histidine residues that activate the nucleophile cysteine. The highly conserved CRD forms six β-strands coordinating two zinc ions, which impart structural stability. This part of the molecule also contains a patch of positively charged residues that bind the negatively charged phosphates of the CoA moiety.

The available crystal structure of human DHHC20 intermediate with 2-bromopalmitate attached to the catalytic Cys^156^ residue provided a first molecular-level explanation for its preference for palmitate over other types of fatty acids [11]. The aliphatic chain was shown to be inserted into a hydrophobic cavity formed by four TM α-helices, and the amino acid residues in the groove interacting with the acyl chain have been identified [11]. It was shown that at the narrow end of the cavity Ser^29^ forms a hydrogen bond with Tyr^181^, which effectively closes the groove. DHHC20′s preference for transferring mainly C16:0 switched towards the increased binding of myristate when the small serine was substituted with a bulky phenylalanine (S29F mutant). In contrast, DHHC20 demonstrated a stronger preference for stearate when the bulky tyrosine was replaced with a small alanine (Y181A) [11]. Notably, Ser^29^ and Tyr^181^ are not conserved across all DHHC proteins; different DHHCs contain two bulky residues, or one bulky and one small side chain, or two small amino acids, at these two homologous positions [27].

Molecular dynamics (MD) simulations are an excellent alternative method employed to examine protein structures in dynamics [29]. It gives especially valuable information for membrane proteins immersed in a lipid environment [30]. To date, human DHHC20 is the only DHHC-PAT that has been studied using MD simulations [31]; it was proposed that hDHHC20 induces a local deformation of the membrane, particularly at the cytoplasm-facing site where the catalytic cysteine is located [32].

In the present study, we perform a series of MD simulations of hDHHC20 and its mutants S29F and Y181A embedded in a hydrated lipid bilayer. These trajectories complement previous investigations [11,31,32] aiming to elucidate the molecular basis of differential S-acylation. We found that a bona fide inner cavity in the DHHC enzyme, gated by a conserved Trp^158^ residue, is only formed when there is an acyl chain inside the protein. The cavity demonstrates ”geometric selectivity” in relation to the lipid it accommodates. We surmise that appropriate positioning of sulfur atoms of both partners of the reaction (the catalytic Cys^156^ of the DHHC enzyme and the acyl-CoA substrate) at the lipid membrane border lies at the basis of the enzyme’s selectivity. We hypothesize that it determines the time of a thioester bond formation, operating as a speed-limiting factor.

In addition, an unexpected phenomenon for membrane proteins was found: a pronounced hydrophilic ridge of TM helix 4 of hDHHC20 exposed to the hydrophobic core of the lipid bilayer. We thus speculate that the transfer of the acyl residue from the acyl-DHHC intermediate to the protein substrate is based on certain DHHC’s specific spatial features. Our results unveil certain previously unknown DHHC enzyme–protein substrate interactions. The obtained experimental data on the S-acylation of SARS-CoV-2′s spike protein are consistent with the molecular modeling results.

## 2. Results

### 2.1. Design of the Study

**Figure 1:**
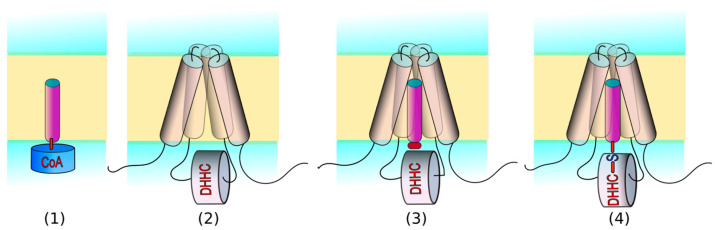
Flowchart of the study. Four system types were simulated in the current research via MD, all immersed in a mixture of POPC:POPE:POPI 6:3:1 mimicking the endoplasmic reticulum (ER) membrane or in a POPC membrane (see Methods): (**1**) Acyl-CoA as an unbound substrate. (**2**) hDHHC20 (wild type (WT) and its mutants S29F and Y181A) are unbound enzymes. (**3**) Proteins bind the substrate analogue (acyl-MEA), but the chemical bond is not formed yet. (**4**) Upon completion of the autoacylation stage of the DHHC cycle, the acyl chain is transferred and covalently linked to the catalytic cysteine’s sulfur atom. In systems of types 2 to 4, we investigated three protein variants: hDHHC20^WT^ and two of its mutants. In systems of types 1, 3, and 4, a range of acyl chain lengths was explored: from C12 to C20. Color code: protein: *gray*; acyl chain: *magenta*; CoA moiety, *blue*; MEA head group, *red*; membrane core: *yellow*; lipids polar heads: *green*; water: *blue*.

In this work, we performed MD simulations of four types of membrane-embedded systems, aiming to describe the autoacylation stage of the DHHC cycle, which is thought to determine the enzyme’s selectivity for acyl chains depending on length. In three of these four cases, systems included acyl moieties to explore the selectivity of the hDHHC20 enzyme and its mutants (Figure 1). The range of acyl chain lengths, each introduced in a separate MD run, was as follows: C12:0 (lauryl), C14:0 (myristoyl), C16:0 (palmitoyl), C18:0 (stearoyl), and C20:0 (arachidonyl). To set up MD simulations, we used both ligand-free (PDB ID: 6BMN) and ligand-bound (inhibited by the covalently-linked 2-bromopalmitate; PDB ID: 6BML) hDHHC20 structures [11]. Two point mutants of hDHHC (S29F and Y181A) were included in the investigation because of their known effect on fatty acid selectivity (see Introduction) [11]. The aforementioned four types of the systems are:

1. Acyl-CoA (C12–C20) in the model membrane is the first DHHC substrate required for the autoacylation step.
2. Free DHHC20 enzyme and two of its mutants (S29F and Y181A) describe the protein in the unbound state, prior to the auto- and transacylation reactions. These calculations provide information on the dynamics of the protein and its binding cavity when no substrate disturbs its interior.

These two systems contain the unbound reaction partners. Since classical MD calculations are not suitable for modeling chemical reactivity, we split the acylation stage into two: immediately before and immediately after the reaction:

3. Within its inner cavity DHHC20 (and the mutants) accommodates acyl-β-mercapto-ethylamine (MEA), an acyl-CoA analogue lacking a large 3′-phosphorylated ADP, without the formation of a chemical bond. This state corresponds to the pre-acylation step, when the substrate analogue has already entered DHHC’s cavity, but has not yet reacted and is thus free to move.
4. Acylated DHHC20 (and the mutants) models the autoacylation product, which is already activated for the transacylation of the substrate protein. In this case, DHHC20 topology was modified to form a chemical bond between the cysteine’s sulfur atom and the fatty acid’s Cα-atom, which restricts the movement of the fatty acid residue.

Systems of types 3 and 4 include all combinations of three protein variants and five acyl chains, yielding 3 × 5 = 15 MD runs in each case. All of the systems built are listed in Table 1, where keywords “Protein” and “Acyl” are used for convenience.

Finally,

5. We performed about 15 in silico mutations to discover novel amino acid substitutions that shift hDHHC20 selectivity towards C18 (Table S1).

Total MD simulation time in this work exceeds 27 µs; details on the systems composition and simulation lengths are summarized in Table 1 and Table S1. Below, the results for systems of types 2 to 5 are presented in detail.

### 2.2. DHHC Avoids a Vacuum: The Central Cavity Is Mostly Occupied

Our simulations of various free acyl-CoA moieties showed that the fatty acid tails insert themselves into the membrane and that the CoA part is exposed to the cytosol, regardless of the length of its acyl chain (series 1 in Figure 1 and Table 1). Their sulfur atoms are located in the ester layer of the membrane and therefore are in an optimal position to react with the DHHC active site’s catalytic cysteine residue (Figure S1), which corroborates earlier findings [31]. However, for autoacylation to be efficient, correct positioning of both sulfur atoms might be required, which is accomplished via the capture of the acyl chain by the DHHC cavity. From the recent crystal structure of hDHHC20, inhibited by covalently bound 2-bromopalmitate (PDB ID: 6BML), one can discover that this substrate analogue is accommodated by a cone-shaped cavity, formed by four TM α-helices folded in a teepee-like manner [11]. The corresponding structure of apo-hDHHC20 (PDB ID: 6BML [11]) contains an empty space, which is well-fitted to capture hydrophobic tails.

**Figure 2:**
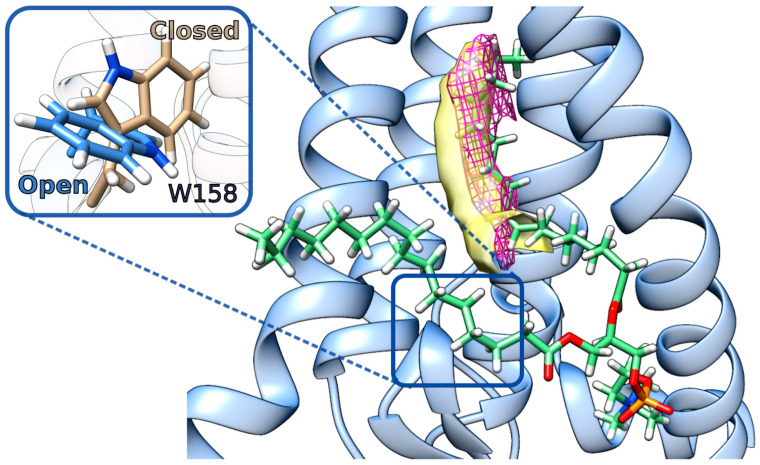
The ligand-free cavity of DHHC20 becomes occupied by a lipid’s alkyl chain in the course of MD. A teepee-like fold of four TM α-helices of DHHC family proteins forms a cavity, which easily accommodates hydrophobic chains—either from its ligand acyl-CoA or from membrane phospholipids (as illustrated in this MD snapshot). The TM helices of hDHHC20 are rendered as a *light blue cartoon*. A single POPC molecule that penetrates the cavity with one of its tails is shown in *stick representation*. A 2Fo−Fc electron density map for PDB ID: 6BMN (*pink mesh*) and a 3D free volume distribution map derived from MD simulation (*yellow surface*) are shown. Inset: Trp^158^ gates the DHHC20 cavity entrance. Two conformations from the MD are superposed: “open” (*blue*; when Trp^158^ does not block the way) and “closed” (*beige*; when Trp^158^ prevents the substrate from entering the cavity). See also Figure S2 for Trp^158^ states dynamics.

In order to explore the properties and free volume of this cavity, we performed MD simulations of the apo-hDHHC20 (and its two mutants) in both a POPC and a mixed POPC:POPE:POPI bilayers (series 2 in Figure 1 and Table 1). Notably, the cavity, which pre-exists in this structure as a sort of “vacuum”, disappears without a delay in the course of MD, manifesting sparse protein packing in its place. This low-density packing area is attractive for hydrocarbon chains, and it soon becomes filled by one of the phospholipids’ acyl chains (see Figure 2). The insertion of acyl chains is dynamic: a lipid tail does not stay inside the cavity during the entire MD simulation time, but it may be replaced by acyl chains from other phospholipids (Figure S2B). Despite such lipid exchange, the cavity remains occupied during most of the MD trajectory (78%; see Figure S2). Both saturated palmitoyl (C16:0) and unsaturated oleoyl (C18:1) chains entered the cavity, without any preference regarding retention time or insertion depth. C16:0 and C18:1 were found inside for 56% and 44% of the “occupied” time, respectively. This preference was not affected by the bilayer type or by the protein variant studied. Our findings corroborate an earlier MD investigation, which had shown that lipid tails of different POPC molecules penetrate the cavity sequentially [31].

The low packing density cavity inside hDHHC20 is screened off from the membrane medium by the conserved Trp^158^ residue, which operates as a gate: it is open when the indole ring is oriented towards the membrane lipids and does not block the way (Figure 2 *inset*, Figure S2). In its closed state, Trp^158^ partially loses contact with Tyr^69^: a hydrogen bond between them exists 58% of MD time as opposed to 90% in an open state.

So, why was the apo-hDHHC20 structure described as possessing an empty cavity? It seems that in fact there is no void space: a close examination of the raw data of the 6BMN entry reveals an unassigned excess electron density inside the cavity (see Figure 2, *pink mesh*), which may be attributed to a fatty acid residue. This cavity is never empty; instead, it may be occupied by a tail of either a phospholipid or an acyl-CoA molecule, which not only enters the cavity, but donates the acyl group to effectuate the autoacylation step of the DHHC cycle.

### 2.3. The DHHC Cavity Determines the Acyl Chain Length Preference

After an initial assessment of the dynamics of the unbound partners, we studied hDHHC20 that binds acyl-CoA (pre-autoacylation stage) or is acylated by the fatty acid residue (autoacylation). The reference structure for these studies was hDHHC20 covalently bound to 2-bromopalmitate via Cys^156^ (PDB ID: 6BML [10]): this molecule was used as a reference to place ligands inside the cavity correctly.

At the pre-autoacylation stage (series 3 in Figure 1), we mimicked acyl-CoA embedding into the hDHHC20 cavity with recourse to an acyl-MEA molecule, which is chemically similar but has a less voluminous “head” compared to the bulky CoA moiety and is therefore optimal for structural studies and modeling. During the initial system assembly, we placed the sulfur atoms of ligands and cysteines in close proximity, at the same time aligning the acyl chains of the ligand and the reference molecule from the 6BML structure. This system was assembled for three DHHC20 variants (WT, S29F, and Y181A) and five acyl chain lengths (acyl = C12, C14, C16, C18, and C20), amounting to a total of 15 systems, followed by 500 ns MD calculations (see Table 1 for details).

**Figure 3:**
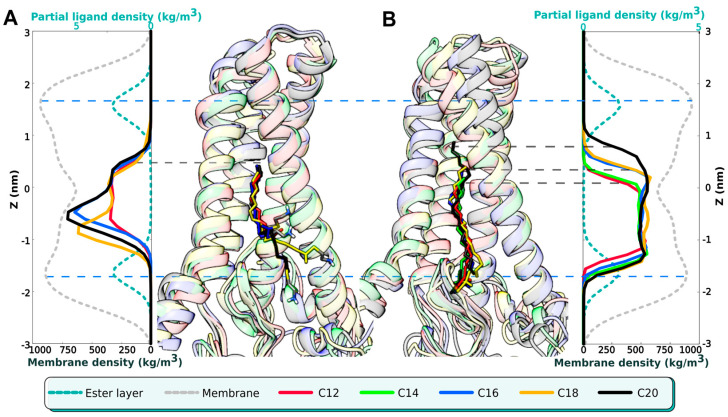
DHHC20 autoacylation: modeling of the pre- and post-acylation states. Two series of MD calculations are summarized in this figure, corresponding to the pre-acylation (**A**) and post-acylation stage (**B**) of the reaction. Medially, snapshots from these calculations are presented: five superimposed structures in each group correspond to MD trajectories with acyl moieties of a specific length (C12, *red*; C14, *green*; C16, *blue*; C18, *orange*; C20, *black*); to the left: hDHHC20 with non-covalently bound acyl-β-mercapto-ethylamines; *to the right*: acylated DHHC20 (see also Table 1 for simulations details). Laterally, the corresponding density profiles derived from MD trajectories and scaled to match graphical representation are displayed. The Z axis is the distance orthogonal to the membrane plane with zero placed at the bilayer center. The lower X axis is the partial density of membrane lipids: total (*gray dotted line*) or ester group (*cyan dotted line*) to mark the border of the hydrophobic layer (*blue dashed lines*). The upper X axis is the acyl chain partial density (coloring as *above*). Note that non-covalently bound acyl chains (*left*) reach a single level (marked with *a single dashed line* at Z ≈ 6 Å), while bound chains (*right*) line up as *a ladder*, as demonstrated by an array of *dashed lines*.

Remarkably, regardless of their initial position, all five acyl-MEA molecules slipped into the DHHC cavity in the same manner: all the acyl chain tips reached the same level of Z ≈ 6 Å (see Figure 3A), which is, apparently, the “ceiling” of the cavity. This might indicate that the DHHC cavity (low packing density area) pulls the acyl chain inside to fill the empty space. The ethylamine head groups are less restricted in movement, and in some cases are seen following the acyl chain, which bends and may even exit the cavity between TM α-helices 1 and 4, exposing itself to the lipid milieu. No such observations were made during simulations of unbound acyl-CoA (series (1) in Figure 1), probably because of its more voluminous and hydrophilic head group. Accordingly, we suggest that with acyl-CoA, no such distortion would occur, and the acyl chain should remain extended.

The post-autoacylation stage (series 4 in Figure 1) was modeled by way of modifying the hDHHC20 topology and covalently attaching the acyl chain to Cys^156^. As performed previously, the fifteen 500 ns MD trajectories were calculated (see Table 1). In this case, the movement of the ligand is restricted, and the chain cannot slip into the cavity up to the ceiling. As a result, the tips of the extended acyl chains from different trajectories, when superposed, line up as a ladder (Figure 3, right panel). The longest molecule (C20) finally breaks the ceiling, formed by Ser^29^, Val^185^, and Ser^217^, and pushes its tip through it.

**Figure 4:**
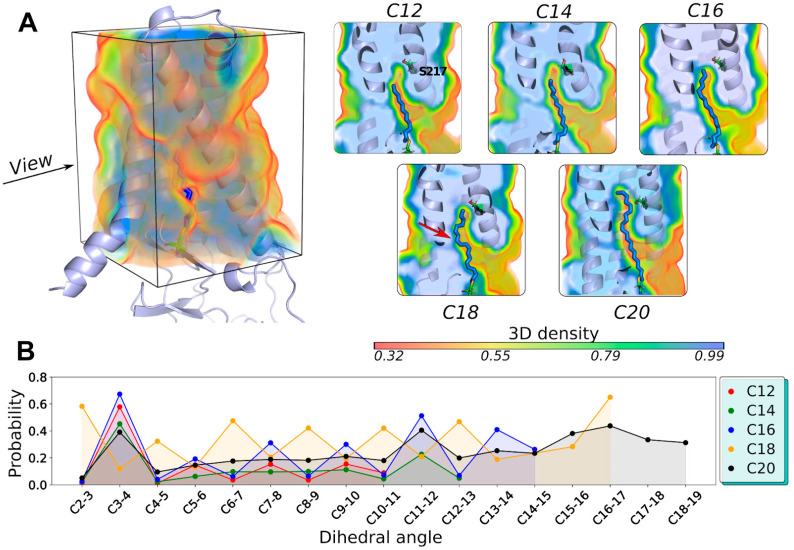
Wild-type hDHHC20 accommodates the C16-acyl best: (**A**) MD-averaged volume occupied by atoms of hDHHC20, acylated with fatty acids of different lengths. ***Left*:** Overview of averaged protein volume (for calculation details, see Methods), which is shown as a *semi-transparent volume* colored from *red* (low-density packing) to *blue* (high-density) in accordance with the scale presented below. Note, the cavity formed around the acyl chain, and compare it with the occupied volume (OV) of the apo-protein, which does not possess such a cavity (Figure S3). ***Right***: Sections of acylated hDHHC20 with corresponding OV. Separate panels show data for C12, C14, C16, C18, and C20 acylation. Note, the low-density packing areas for suboptimal chain lengths C12 and C14 just below the subscribed Ser^217^ residue; optimal packing of C16; kinked C18 acyl chain (*red arrow*); and C20 breaking the “ceiling” of the cavity. (**B**) Deviation of the acyl chains from the *all-trans* conformation while bound inside the hDHHC20 cavity. The plotted value is the integral probability density that deviates from the fixed dihedral angle of 180° by more than 75° (for full analysis, see Figure S4). Note that C12 and C14 substrates are the least distorted, C16 has moderate values, while C18 is the most tense. C20 is more relaxed again since it breaks the “ceiling” and finds some room to straighten.

In trajectories with covalently acylated hDHHC20 (series 4 in Figure 1 and Figure 3B), there is an area of low packing density above the acyl chain tips if the acyl chain length is “suboptimal” (C12 and C14 for DHHC20^WT^; see Figure 4A). C16, which has the optimal length for hDHHC20 [11], fits the cavity best: it occupies the entire space and keeps the chain extended. C18 seems to occupy the same space (the tip of the chain reaches the same level), but its acyl chain kinks 3 to 4 times, which pushes the cavity walls apart and leads to the deviation of the chain conformation from the *all-trans* state (Figure 4B and Figure S4). In the C20-acylated hDHHC20, the acyl chain is inappropriately long, and the only way to accommodate it inside the cavity is to break the “ceiling” and extend the cavity beyond residues Ser^29^, Val^185^, and Ser^217^, which form this natural barrier. Thus, alkyl chains exceeding optimal length break through the bottleneck, disrupting protein contacts.

### 2.4. hDHHC20 Mutants S29F and Y181A Modify the Free Space of the Cavity

The same study that described the spatial structure of hDHHC20 reported that its S29F mutant exhibits a shift in fatty acid selectivity towards shorter acyl chains (C12 and C14, as opposed to C16 for WT), while the mutant Y181A equally accommodated C16 and C18 [11]. Our structural analysis suggests that these mutations decrease and increase, respectively, the free space to accommodate the acyl chains. We performed MD simulations of these two mutants in addition to hDHHC20^WT^ to test this hypothesis in dynamics (series (4) in Figure 1 and Table 1). An array of acyl chain lengths enabled us to thoroughly analyze how these proteins accommodate their substrates, and which fits best.

**Figure 5:**
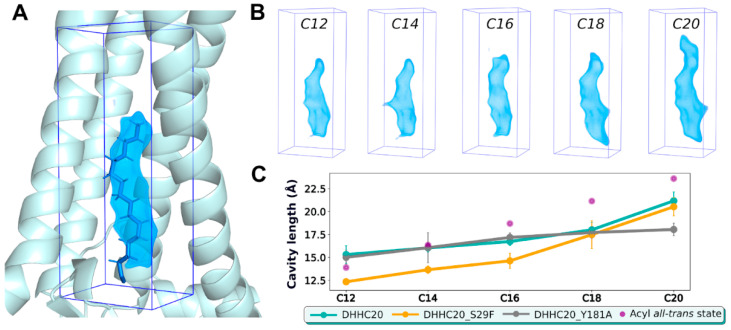
Volume and length of the cavity inside acylated hDHHC20 and its variants: (**A**) A snapshot of palmitoylated hDHHC20 (C16) from MD calculation. *Semi-transparent blue volume* contours low-density protein area, induced by the acyl chain (data averaged over MD trajectories; see *Methods* for details). (**B**) Corresponding volumes for DHHC20/C12 to C20 trajectories. (**C**) Dependence of the cavity length on the length of the acyl chain bound (C12 to C20) for hDHHC20^WT^ and its two mutants, S29F and Y181A. Lengths of the extended acyl chains (*purple dots*) are provided for comparison (see *legend*).

To perform this analysis, we calculated volumes (Figure 5A,B) and lengths (Figure 5C) of the cavities that acyl chains occupy inside the protein, averaged over their respective MD trajectories. The cavity appears to be a “cast” of the acyl chain, and it would seem that there are no interesting effects to be found based on this observation. Counterintuitively, however, there is a difference in how the cavity’s length depends on the acyl chain length (Figure 5C). One can see that the cavity’s length barely changes while the chain lengthens from C12 to C18, but then abruptly increases in the case of a C20 chain. This confirms the results presented in Figure 4A: for “suboptimal” lengths, the cavity still has some void space above the acyl chain tip, providing room to slip into.

Our analysis of the hDHHC20 mutants reveals that Y181A possesses a deeper cavity for all acyl chain lengths: hence, the higher affinity to the C18-acyl (Figure S5). Interestingly, this substitution adds room not just above the cavity (in the “ceiling”), but, instead, on the side, making the acyl chain’s tip twist to enter this new volume. In contrast, S29F’s cavity becomes considerably shorter (Figure 5C) due to the increased size of residue F29′s side chain, truncating the cavity and shifting the selectivity towards shorter chains.

### 2.5. In Silico hDHHC20 V185G Mutant Exhibits C18-Selectivity

**Figure 6:**
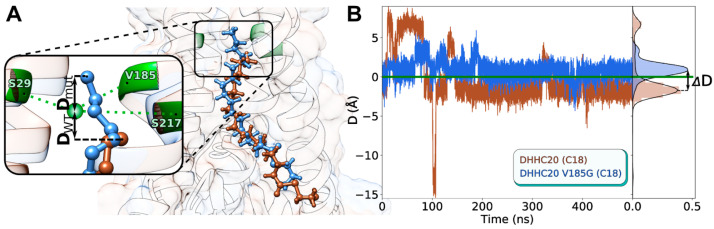
MD simulations suggest that hDHHC20^V185G^ should exhibit a C18-selectivity shift: (**A**) The “ceiling” of the hDHHC20′s cavity is formed by a triad of residues: Ser^29^, Ser^217^, and Val^185^ (here substituted with glycine). Two superimposed structures in complex with the C18-substrate are presented: hDHHC20^WT^ (*brown* substrate) and hDHHC20^V185G^ (*blue* substrate). The tip of the acyl chain in the latter structure surmounts the “ceiling”, which is described by the D parameter: the distance between the terminal carbon atom and the center of mass of the “ceiling” triad (see *inset* for a more illustrative explanation). (**B**) Dynamics of the D parameter for the two said systems. D = 0 is the level of the “ceiling”. *To the right* are probability density functions for these D distributions, allowing the calculation of ⟨D⟩^WT^= −1.7 ± 0.7 Å, ⟨D⟩^V185G^ = 0.9 ± 0.7 Å and ΔD = 2.6 Å.

Having established the reason for selectivity change in the previously reported hDHHC20 mutants Y181A and S29F [11], we set a goal to predict new mutant variants with a selectivity shift towards C18 and assess them in silico. Our structural analysis reveals that the cavity’s “ceiling” is formed by a triad of residues: Ser^29^, Ser^217^, and Val^185^ (Figure 6A), and that a decrease in their cumulative volume should provide additional room for two extra methylene groups of a C18 substrate. Notably, the authors of the reported C18-selective hDHHC20 variant [11] implemented the Y181A substitution instead of mutating any of the triad residues, which resulted in a curved cavity with extra volume located to the side of the cavity axis.

To test this hypothesis, we introduced in silico point mutations to the hDHHC20 structure and performed MD calculations with C18-MEA as substrate in a lipid bilayer. For the full list of the tested mutants, see Table 1 and Table S1. The effect of the mutations introduced was assessed with recourse to parameter D, corresponding to the relative position of the acyl chain’s tip (the terminal carbon atom) with respect to the “ceiling” residues’ center of mass (see Figure 6A for D definition and Figure 6B for dynamics). Negative D means that the acyl chain tip is located “below” the ceiling, while positive D indicates that the ceiling was surmounted (due to increased free space and the lengthening of the cavity); thus, a positive D shift accompanying the mutation suggests that a more voluminous substrate might be accommodated inside the cavity. Figure 6B illustrates the corresponding selectivity shift for hDHHC20^V185G^: while D = −1.7 ± 0.7 Å for hDHHC20^WT^, it shifts to 0.9 ± 0.7 Å for the V185G mutant. We imply that the observed ΔD = 2.6 Å (Figure 6B, *right panel*) should be enough to accommodate the C18 substrate and predict a selectivity shift for this mutant variant towards the stearoyl chains.

Importantly, none of the point mutations to alanine, V185A, S29A, or S217A, demonstrated a positive ΔD (see Table S1): either this volume increase is too small, or it is compensated by the protein’s adaptive TM helices, which intersect in this area and form a “folding core”. Similarly, substitution L213A, introduced right above the ceiling triad, did not work either. More successful were double mutants: S29A+V185A worked partially, exhibiting two interconvertible states, one with ΔD = 4.7; V185G+S217A, which had been assessed prior to making the single V185G substitution, worked perfectly (D = 1.6 ± 0.5 Å, Table S1). Finally, we tried to move apart the teepee-like helical structure of DHHC20, introducing the “off-ceiling” mutations to voluminous residues: Y33W, F62W, A32L, F58W, F184W, A186L, L213F, and V185I. None of these had an effect.

### 2.6. Human DHHC20 Might Recognize the Spike Protein of SARS-CoV-2via Hydrophilic Interactions between Their Transmembrane Regions

**Figure 7:**
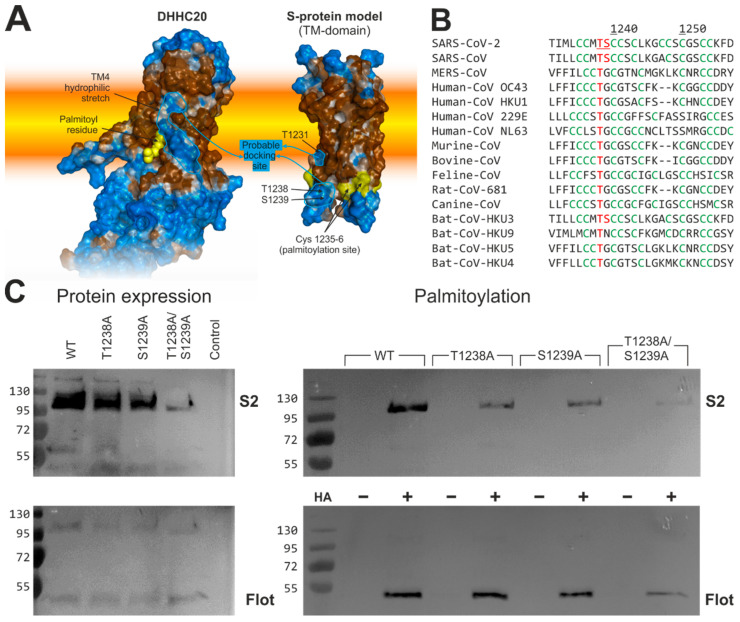
The probable role of hydrophilic patterns on hDHHC20 and S protein TM-domain surfaces in the Spike acylation: (**A**) Hydrophobic/hydrophilic properties distribution on the hDHHC20 enzyme (*left*; PDB ID: 6BMN) and SARS-CoV-2 S-protein (*right*; computational model; see *Methods*) TM-domain surfaces; the membrane is schematically shown as a *yellow-orange slab*. Hydrophilic and hydrophobic areas are colored *blue* and *brown*, respectively; calculation was performed in accordance with the Molecular Hydrophobicity Potential formalism [35,36]. (**B**) Alignment of the Spike proteins of various human and animal coronaviruses at the boundary between the transmembrane and cytoplasmic domains. The cluster of acylated cysteines is in *green*, the two hydroxyamino acids Thr^1238^ and Ser^1239^, which were replaced with Ala in the S protein of SARS-CoV-2, are in *red*. Note that Thr^1238^ is completely and Ser^1239^ partially conserved in S proteins from other coronaviruses. (**C**) Acylation analysis of WT and mutant S protein expressed in 293T cells. ***Left panel*:** To test for protein expression 10% of the cell lysate was removed. ***Right panel*:** The remainder of the lysate was divided into two aliquots, one not treated (−HA) and one treated with hydroxylamine (+HA) to cleave cysteine-bound fatty acids before pulling down proteins with a free SH-group. Samples were subjected to Western blotting with antibodies against the S2 subunit of SARS-CoV-2′s Spike and, subsequently, with those against the endogenous protein flotillin (Flot). Molecular weight markers are shown on the left of each blot.

Analysis of hydrophobic/hydrophilic properties of the hDHHC20 surface reveals a prominent hydrophilic stretch in TM helix 4 precisely where it faces the membrane’s hydrophobic core, which is unusual for TM domains [33,34] (Figure 7A, *left*). This feature of hDHHC20 is stable over the course of MD simulations (data not shown), and we, therefore, assume that this peculiarity may correspond to the docking site, where the DHHC protein substrates initially get associated in order to be acylated by this enzyme. Many of hDHHC20′s substrates contain hydrophilic amino acids in the vicinity of their acylation sites (data not shown), including the Spike (S) protein of SARS-CoV-2, as assessed on the basis of a 3D model of its transmembrane domain (Figure 7A, *right*). 

The Spike protein of SARS-CoV-2 is acylated at a cluster of 10 cysteines, located in the N-terminal portion of the cytoplasmic domain, mainly by hDHHC20. The model of the Spike exhibits three hydrophilic amino acids, Thr^1231^, Thr^1238^, and Ser^1239^, at the surface of the molecule near the first two cysteines of the acylated cysteine cluster. This cluster of acylated cysteine residues is not a unique feature of the SARS-CoV-2 Spike; in fact, it is also present in Spike proteins of human pathogens MERS-CoV and SARS-CoV-1, in those of four human “common cold” coronaviruses OC43, HKU1, 229E, and NL63, as well as in Spike proteins of various pathogenic animal coronaviruses and viruses found in bats, a wildlife reservoir of a wide variety of coronaviruses (Figure 7B). Sequence comparison also revealed that a Thr is present in all of the aforementioned Spike proteins at a position corresponding to Thr^1238^ in SARS-CoV-2′ Spike, while a Ser is present in those of SARS-CoV-1 and bat coronavirus Bat-CoV-HKU3. 

To analyze whether Ser and Thr positively affect acylation, they were substituted with Ala, either individually or in combination. The resulting proteins were expressed in 293T cells, which were lysed 48 h after transfection. A 10% aliquot was used to compare the expression levels of wild-type and mutant Spike. The remaining sample was equally split and subjected to Acyl-RAC (resin-assisted capture) to determine whether the acylation of S is reduced in any of the mutants. Acyl-RAC exploits thiol-reactive resins, which capture SH-groups newly liberated by hydroxylamine cleavage of thioester bonds (+HA in Figure 7C). The other half of the aliquot was treated with Tris–HCl instead of hydroxylamine to assess the binding specificity of the resin (−HA). All samples were then subjected to Western blotting with a monoclonal antibody against the S2 subunit and subsequently with an antibody against the endogenous palmitoylated protein flotillin, which serves as a control for loss of material during sample preparation. 

The results revealed that +HA signals of S protein single mutants T1238A and S1239A are much weaker compared to S^WT^ and barely detectable for the S protein double mutant T1238A/S1239A, indicating that acylation is greatly reduced. Note, however, that the expression levels of the mutant S are also diminished, although, to a lesser extent. The lesser protein expression demonstrated the double T1238A/S1239A mutant, while protein expression of both single mutants reduced just slightly and cannot explain the great decrease in their palmitoylation level. It has recently been shown that the removal of acylation sites destabilizes the trimeric Spike and causes its premature degradation [26]. Therefore, we assume that the weaker bands of Spike T1238A/S1239A could be attributed to the same mechanism: reduced acylation causing degradation of the spike. Thus, we propose that Thr^1238^ and Ser^1239^ may be part of the motif that is recognized by hDHHC20 to allow its efficient acylation, presumably because it binds to the hydrophilic ridge in TM helix 4 of hDHHC20.

## 3. Discussion

So far, little is known about molecular mechanisms that enable DHHC acyltransferases to contribute to acyl chain heterogeneity within a protein substrate. Differences in the fatty acid selectivity profiles of cellular DHHC enzymes were previously uncovered using the click chemistry approach [20]. However, the structural molecular basis of such selectivity is still poorly understood. Here, we used a molecular modeling platform to explore structural and dynamic features determining the course of the first autoacylation step of the S-acylation reaction implemented by hDHHC20, an important member of the DHHC acyltransferase family. The approach is based on atomistic MD simulations of the membrane-bound enzyme, several of its mutants and their complexes with various ligands, along with detailed mapping of physicochemical properties of the hDHHC20 inner cavity, which accommodates lipid substrates. 

For the first time, we propose the principle of geometrical and physicochemical selectivity in the inner cavity of DHHC-enzyme as the main criterion underlying the acyl-chain length binding preferences. The initial hypothesis is based on the 3D structure of hDHHC20 obtained in a complex with covalently bound 2-bromopalmitate [11] and a series of MD simulations of free Acyl-CoAs (C8:0, C16:0, and C22:0) carried out in POPC bilayer [31] were now tested using long-range (microsecond) MD simulations mimicking four different steps of the autoacylation reaction: (1) free Acyl-CoA, (2) free DHHC-enzyme, (3) DHHC-enzyme with (non-covalently) bound/immersed in the inner cavity acyl-CoA substrate analogue, and (4) DHHC-enzyme with a covalently bound acyl chain. Moreover, a unique Molecular Hydrophobicity Potential algorithm showed the complementarity of the physicochemical properties of the inner cavity of hDHHC20 and the lipid substrate of a particular length. Thus, based on a wide panel of lipid substrates and WT/mutant hDHHC20 enzymes, we performed a comprehensive investigation of acyl-CoA–DHHC-enzyme interactions and construct for the first time a V185G hDHHC20 enzyme that specifically transfers C18:0.

Finally, the MHP approach revealed for the first time an unusual hydrophilic ridge at the surface of hDHHC20 exposed to the hydrophobic lipid environment. We showed that this hDHHC hydrophilic region is a very plausible docking site for the SARS-CoV-2 S-protein, and experimentally managed to reduce the S-protein’s massive fatty acylation via site-directed mutagenesis of the complementary hydrophilic patch on its surface. We will discuss these various achievements in more detail below, but before we discuss the implications of our study, we would like to address some limitations of our approach.

In principle, the identification of the mechanisms of the lipid selectivity of DHHC enzymes would certainly require a thorough comparative analysis of at least several proteins differing in lipid substrate preference. However, the conclusions in this work are derived from one example only, hDHHC20, for which the 3D structure is resolved, and the effects of a number of point mutations within the cavity on lipid selectivity are described. This choice is due to our failure to build homology models of all DHHC members, as sequence divergence within this enzyme family is very high. However, the principle of the S-acylation reaction and, more particularly, its autoacylation step, implies that the delivery of the lipid substrate to the enzyme’s active site requires strictly determined positioning of the catalytic cysteine in relation to the lipid bilayer border. The accuracy with which this problem can be solved using modeling methods critically affects the precision wherewith the mechanism of the enzymatic process as a whole would be predicted. Therefore, at this stage, we decided to refrain from large-scale comparative structural analysis of the cavities of an entire set of enzymes, and to only study the behavior of membrane-bound hDHHC20 in the presence of various ligands in thorough detail.

It should be noted that MD calculations were performed for a full-sized hDHHC20 molecule embedded into an explicit hydrated lipid bilayer. At the same time, primary attention during data analysis was focused on the behavior of the enzyme’s lipid-binding site, an internal cavity formed by its four TM domains. On the one hand, this allowed us to study in detail the two most important stages of the S-acylation reaction occurring directly in the active center, immediately before and after the covalent attachment of the alkyl chain to the catalytic cysteine. On the other hand, this narrow focus has its drawbacks. In particular, we did not consider the potential allosteric effects and collective movements in the system, which can affect protein activity. This issue, apparently, could be especially significant for the DHHC acyltransferases, where the functional characteristics strongly depend on the fine ligand-mediated dynamic regulation of the geometric parameters of the lipid-accepting groove and the positioning of the active center relative to the membrane border. In addition, the heterogeneous water–lipid environment of the enzyme can also contribute to its structural and functional characteristics, since both the TM-domains and membrane–water interface are involved in the enzymatic process. In the future, we plan to consider these issues, but this is the topic of a separate study.

Here, we computationally evaluated the binding of different acyl-CoA forms with C12:0, C14:0, C16:0, C18:0, and C20:0 fatty acid chains as lipid substrates to membrane-embedded human hDHHC20, its experimentally tested S29F and Y181A mutants, along with a series of 15 mutants designed in silico. We have found that the lipid membrane is an essential factor that influences the formation of the enzyme’s catalytic inner cavity: this cavity is only formed properly after the insertion of a fatty acid from an adjacent phospholipid or from an acyl-CoA substrate.

Another volume of results prompts us to consider the mechanism whereby the lipid specificity of the acylation reaction is achieved. In principle, it would be conceivable that only acyl chains of an appropriate length would be taken up into the hydrophobic tunnel and then transferred to the cysteine of the DHHC motif. However, we found that all the acyl chains studied here can enter the hydrophobic tunnel, but the distance between the sulfur atoms in acyl-CoAs and in the cysteine of DHHC is shortest in the case of Pal-CoA. We hypothesize that the efficiency of autoacylation crucially depends on the precise mutual positioning of these two atoms. We found that the length of the favorably accommodated fatty acid residue depends on the cavity configuration and, in particular, on its ceiling. In cases when the acyl chain length equals that of the cavity, autoacylation proceeds with the best output. In other cases (if the acyl chain is longer or shorter than the cavity), the distance between the said sulfur atoms increases and autoacylation yield drops.

Our results thus explain why a single peak on lipid substrate preferences’ curves of hDHHC20 and its mutants was observed in biochemical experiments [11]. Although a rather broad spectrum of lengths of fatty acid tails can be accommodated, there is only one that fits best. However, if an inappropriately long fatty acid residue is eventually attached to the enzyme, its aliphatic tail has to unfavorably twist to be accommodated inside the cavity, disrupting the necessary hydrophobic contacts with corresponding amino acid residues within the groove.

Apart from a merely geometric fit, we show that physicochemical complementarity also exists: an almost perfect match of the hydrophobic/hydrophilic properties of the ligand and the enzyme’s cavity is only observed for the hDHHC20^WT^–C16 pair (Figure S6). Other acyl chains immediately disturb the optimal fit. The totality of modeling data confirms the lipid selectivity of DHHC acyltransferases implemented at the first autoacylation step of the S-acylation reaction. Not only does the proposed model explain the lipid preferences of the previously described S29F and Y181A mutants, but it also permits in silico screening of other amino acids located at the ceiling of the tunnel. Replacement of Val^185^ with a Gly allows C18 to slip into the tunnel in a conformation suitable for autoacylation (Figure 6); hence, residue 185 (together with residues 29 and 181) might regulate the fatty acid preference of hDHHC20.

Based on these results, we can make several conclusions. First, the inner cavity’s length is really the crucial factor that should determine the fatty acid specificity of the S-acylation reaction. Notably, V185G is the first mutant that not only simply adds the ability for hDHHC20 to bind C18 together with C16 (like the previously described Y181A mutant did), but really shifts the hDHHC20′s preferences of binding C18 instead of C16. Actually, when this prediction is confirmed in a “wet” experiment in the future, it would be a very strong argument against an alternative hypothesis that just the Pal-CoA/Stear-CoA proportion present in the vicinity of an acyltransferase/protein substrate or just within a cell mainly affects the lipid specificity. Second, the data obtained bring us closer to understanding mechanisms of specific S-stearoylation (binding primarily C18:0) of, e.g., Influenza C virus hemagglutinin-esterase-fusion glycoprotein or Newcastle disease virus F protein. Homology model building based on the hDHHC20′s V185G mutant helps find the exact DHHC-enzymes preferentially transferring stearate in cells. Blocking those enzymes using specific inhibitors may be a promising strategy to struggle with those viruses.

Our MD simulations also revealed two different rotamers of Trp^158^ in the ligand-free and ligand-occupied form of hDHHC20. Trp 158 is located two amino acids downstream of the catalytic DHHC motif at the entrance of the hydrophobic tunnel. The side chain of Trp is turned outwards away from the tunnel’s entrance in the ligand-occupied state, but turned inwards in a ligand-free state, blocking the entrance to the tunnel. Since this Trp is conserved at an equivalent position in every DHHC enzyme, it might act as a general gate for the hydrophobic tunnel and, in consequence, might regulate the enzyme’s activity.

In addition to detailed mapping of the properties of the binding cavity and its congruence with lipids differing in acyl chain length, we also detected some putative structural signals in hDHHC20 for binding a protein substrate. There are unusual hydrophilic regions at the outer surface of the teepee-like structure formed by four TM-α-helices: in particular, a prominent hydrophilic stretch is clearly seen in TM helix 4 (Figure 7A). Since the presence of a hydrophilic surface is highly unfavorable within the hydrophobic environment of a lipid bilayer [33,34], we assume that this surface might serve as a docking point of a potential substrate.

The S protein of SARS-CoV-2 is currently one of the most important and intriguing substrates for hDHHC20. Therefore, we decided to check whether there is a certain “response” motif on the corresponding part of the S-protein surface that can facilitate intermolecular recognition and interaction. The analysis of the 3D model of the S-protein trimer’s TMD constructed by us for the first time revealed a reciprocal polar pattern, mostly constituted by Thr^1238^ and Ser^1239^. The threonine is conserved in S-proteins of animal and human coronaviruses (Figure 7B), suggesting a common mechanism employing interaction of the polar surfaces of DHHC and Spike inside the membrane (Figure 7A). Finally (and most importantly), substitutions that disrupt this hydrophilic cluster in Spike led to decreased S-acylation of this protein. Further biochemical and structural studies are required to identify all the amino acids that mediate this interaction. 

The information obtained in the present study might one day facilitate the design of peptide-based compounds that bind to hDHHC20 with a high affinity and block its interaction with the viral Spike protein. In general, currently licensed antiviral drugs are directed against targets encoded by the virus, but treatments, due to the high mutation rate of RNA viruses, rapidly generate drug-resistant variants that circulate in the population. In contrast, a cellular target has a low potential to develop resistance, especially if the drug is used locally (respiratory tract) and over a relatively short period of time (1–2 weeks) [25]. Such specific inhibitors of hDHHC20–Spike interactions could contribute toward overcoming the devastating pandemic of COVID-19. They might even become part of pandemic preparedness plans since Spike proteins of all coronaviruses are acylated at cysteine clusters, which have hydroxy amino acids in their vicinity. In addition, although the number of DHHCs differs between vertebrates, hosts of coronaviruses (including bat, pangolin, and camel) encode a DHHC20 protein, which exhibits 86–92% amino acid identity to human DHHC20. Three-dimensional structural alignments showed that the amino acid exchanges are unlikely to affect the catalytic mechanism of the enzyme. Nevertheless, once a promising drug against hDHHC20 has been developed, its efficiency in the various hosts has to be determined experimentally [24].

So far, however, the only compounds inhibiting all DHHC-proteins that have been identified often also inhibit other enzymes employed in lipid metabolism and hence possess high cytotoxicity. Since the autoacylation step is presumably essential for most DHHC-catalyzed reactions, a new lipophilic inhibitor should be specifically targeted to the hydrophobic cavity of hDHHC20 or at least to the cavity of different DHHCs that prefer palmitate over stearate. Although at least HA of Influenza A virus (as well as a number of other viral glycoproteins [17]) is acylated by both stearate and palmitate, the attachment of palmitate was shown to be more important for virus replication [37,38]. Thus, the design of a specific inhibitor of the DHHC enzyme predominantly transferring palmitate will be an advanced step in modulating the activity of specific DHHC enzymes and would be very useful as part of an antiviral strategy.

## 4. Materials and Methods

### 4.1. Molecular Dynamics Simulations

#### 4.1.1. Systems Setup

The starting model for hDHHC20^WT^ simulation (apo-state) was taken from the available crystal structure (PDB entry 6BMN) [11]. hDHHC20^S29F^ and hDHHC20^Y181A^ mutant models were created by standard mutagenesis option of the Pymol software v. 2.5.0 (Schrödinger, Inc., NY, USA) [39]. The X-ray structure of hDHHC20 with covalently bound inhibitor 2-bromopalmitate (2-BP) (PDB entry 6BML [11]) was used to generate the starting structures of acylated proteins, in which alkylated Cys^156^ was modified via the attachment of a fatty acyl group as a thioester. Both bound and unbound acyl chains were aligned to the X-ray 2 BP position.

Protein structures were embedded in a pre-equilibrated bilayer (144 lipid molecules per monolayer) using the in-house software framework written in C++ and Python. To construct a mixed bilayer, the lipids of different types were randomly placed at the nodes of the regularly spaced square grid preserving the relative concentrations for each leaflet independently. Following the protein insertion, the overlapping phospholipids were removed. In order to generate a physiologically relevant membrane, we used the heterogeneous mixture of zwitterionic 1-palmitoyl-2-oleoyl-sn-phosphatidylcholine (POPC) and 1-palmitoyl-2-oleoyl-sn-phosphatidylethanolamine (POPE), and anionic 1-palmitoyl-2-oleoyl-sn-phosphoinositol (POPI) lipids at a ratio of 6:3:1. It mimics the membrane of endoplasmic reticulum (ER) [40], where many DHHC family proteins are localized [5]. In addition, since the DHHC20 species is also localized in the plasma membrane, we conducted a series of MD simulations in a pure POPC bilayer.

#### 4.1.2. MD Simulation Parameters

MD simulations were performed with the GROMACS (KTH-Royal Institute of Technology, Stockholm, Sweden) software package version 2020.4 [41] using the CHARMM36 force field parameters [42,43,44,45,46]. An integration time step of 2 fs was used and 3D periodic boundary conditions were imposed. The 12 Å cutoff radius was defined for the Coulombic and van der Waals interactions. Long-range electrostatic interactions were handled using the particle mesh Ewald (PME) method [47]. An explicit solvent model was used (TIP3P [48]) and Na^+^ and Cl^−^ ion parameters for counter ions were used [49]. The ionic strength of the solvent corresponded to 150 mM NaCl. Simulations were performed at 310 K temperature and 1 bar pressure maintained using the V-rescale [50] and the Parrinello–Rahman [51] algorithm with 1.0 and 0.1 ps relaxation parameters, respectively. The semi-isotropic pressure coupling in the bilayer plane and along the membrane normal was used in the simulations. Before the production runs, all systems were minimized over 5000 steps using a conjugate gradients algorithm, followed by heating from 5 to 310 K over 0.5 ns, in which internal coordinates of the protein and ligand heavy atoms were restrained. Protein along with membrane lipids and solvent molecules were coupled separately. Trajectory length ranged from 100 ns to 1 μs. All simulations performed are listed in Table 1 and Table S1. Multiple MD runs with a small increment in the length of the acyl chain (-C_2_H_5_) for each protein–lipid substrate system give satisfactory sampling for the states.

#### 4.1.3. MD Data Analysis

Configurations of the systems extracted from MD trajectories were centered on the protein molecule and analyzed with a timestep of 10–100 ps using original GROMACS and custom utilities. Protein and lipid density profiles, atomic distances, and coordinates were extracted using *gmx density*, *gmx dist,* and *gmx traj* utilities, respectively. Intermolecular contacts, including hydrogen bonds, were calculated using GROMACS (*gmx hbond*), PLATINUM (Laboratory of Biomolecular Modeling, Shemyakin-Ovchinnikov Institute of bioorganic chemistry, RAS, Moscow, Russia) [36], and original in-house software. Molecular graphics were rendered using PyMOL v. 2.5.0 (Schrödinger, Inc., New York, NY, USA) [39] and UCSF Chimera package (UCSF RBVI, University of California, San Francisco, CA, USA) v. 1.11.2 [52].

To perform free volume calculation a regularly spaced rectangular mesh 15 × 9 × 8 Å in size measuring 50 × 30 × 30 cells in each direction is placed so that X axis of local coordinate system (CS) would pass through the centers of mesh faces parallel to YOZ plane and the coordinate system origin would be on the lower YOZ face center. Local CS origin coincides with the center of mass (CoM) of CA atoms in DHHC residues 72, 156, and 23. Its X axis passes through the CoM of CA atoms in residues 33 and 185. CA atom of residue 72 lies in the CS XOY plane. The mesh origin was shifted relative to the local CS to exclude free volume outside the DHHC cavity. The shift vectors were adjusted for each MD system separately. For each trajectory frame protein and surrounding molecules are oriented as described above, and 3D distributions of a free volume (FV) accessible to a center of probe sphere with radius = 1.4 Å (CH_3_/CH_2_ group equivalent) are calculated. Initially, all mesh vertices had a weight of 0. During FV estimation each van der Waals (vdW) sphere associated with an atom was checked against nearby mesh vertices. All vertices lying inside a given vdW sphere were assigned weights equal to 1. FV distributions were then averaged over the trajectory. Occupied volume (OV) for each cell was calculated as follows: OV = 1 − FV. The data thus collected were used for visualization (Figure 4A, Figure 5A and Figure 6A,B) and estimation of the cavity height and volume, which were conducted as follows: all cells where occupancy time was less than 50% were excluded, connected components of FV mesh cells in 3D space were identified, and the largest component was selected. The cell count of the largest component was then multiplied by the cell volume, and the size of its bounding box along X direction was calculated (Figure 5C), providing estimated volume and height of the cavity.

Fatty acid tail dihedral distributions were estimated as follows. Dihedral angle distributions for each four consecutive covalently bound carbon atoms in the fatty acid chain forming a dihedral were calculated over the entire trajectory in steps of 100 ps (5000 frames). All values varied from 0 to 180°. Normalized histograms over the entire trajectory for each dihedral were calculated to make Figure S4 and estimate probability density functions (Figure 5B). The latter were integrated from 0 to 105° to estimate the probability of finding a dihedral in the range.

### 4.2. Spike Protein Transmembrane Domain Modeling

The model of the spike protein’s transmembrane domain (TMD) was created in Modeller 9.19 [53]. The trimer included residues 1212 to 1234 and was built based on the TMD of tumor necrosis factor receptor 1 (PDB ID: 7K7A [54]). Small unordered sequences were added upstream and downstream of the TMD also using Modeller 9.19, and the final model eventually included residues 1208 to 1238. The trimer was inserted in a POPC bilayer, and energy minimization and equilibration were performed in GROMACS, after which an MD trajectory was calculated. These calculations were relied on to refine and fine-tune the packing of the model TMD trimer, for which purpose Pymol 2.5.0 and UCSF Chimera 1.14 were used. The final model was probed for stability in a model POPC bilayer over the course of a 1 μs MD trajectory and proved to be highly stable. Ser1239 was appended manually in Pymol 2.5.0.

### 4.3. Mutagenesis, Expression, and Acylation Analysis of the S Protein of SARS-CoV-2

The human codon-optimized spike gene of SARS-CoV-2 was synthesized; PCR was amplified from the plasmid and cloned into a pCAGGS expression vector using XbaI and XhoI restriction sites. Both threonine and serine at positions 1238–1239 were substituted with alanine, either individually or in combination using the quick-change site-directed mutagenesis kit from Stratagene (an Agilent Technologies Division, Santa Clara, CA, USA, #200515 www.stratagene.com, accessed on 2 May 2022). All mutations were confirmed by sequencing. Plasmids were transfected into 293T cells using Lipofectamine 3000 in accordance with the manufacturer’s instructions. S-acylation of Spike was analyzed using the Acyl-RAC assay [55]. Transfected cells were washed with PBS and lysed in 500 µL buffer A (0.5% Triton-X-100, 25 mM HEPES (pH 7.4), 25 mM NaCl, 1 mM EDTA, and protease inhibitor cocktail). Disulfide bonds were reduced by adding Tris (2-carboxyethyl) phosphin (TCEP, Carl Roth, Waltham, MA, USA) to a final concentration of 10 mM; samples were incubated at room temperature for 30 min. Free SH-groups were blocked by adding methyl methanethiosulfonate (MMTS, Sigma, St. Louis, MO, USA, dissolved in 100 mM HEPES, 1 mM EDTA, 87.5 mM SDS) to a final concentration of 1.5% (*v*/*v*) and incubated for 4 h at 40 °C. Subsequently, 3 volumes of ice-cold 100% acetone were added to the cell lysates and incubated at −20 °C overnight. Precipitated proteins were pelleted at 5000× *g* for 10 min at 4 °C. Pelleted proteins were washed five times with 70% (*v*/*v*) acetone, air-dried, and then re-suspended in 1 mL binding buffer (100 mM HEPES, 1 mM EDTA, 35 mM SDS). Then, 20 to 30 µL of the sample was removed to check for total protein expression by Western blotting. The remaining lysate was divided into two aliquots. One was treated with hydroxylamine (0.5 M final concentration, added from a 2 M hydroxylamine stock adjusted to pH 7.4) to cleave thioester bonds. The other aliquot was treated with 0.5 M Tris-HCl pH 7.4. Then, 30 µL thiopropyl agarose beads (creative biomart), which had been washed with Millipore water, were added at the same time to capture free SH-groups. Samples were incubated with beads overnight at room temperature on a rotating wheel. The beads were then washed 5× in binding buffer and bound proteins were eluted from the beads with 2× non-reducing SDS-PAGE sample buffer for 5 min at 95 °C. Samples were then subjected to SDS-PAGE and immune-blotting using a monoclonal antibody against the S2 subunit of the spike (GeneTex, Irvine, CA, USA # GTX632604, 1:3000) and anti-mouse (Bio-Rad Laboratories, Hercules, CA, USA, 1:2000) secondary antibody coupled to horseradish peroxidase. After washing, signals were detected by chemiluminescence using the ECLplus reagent (Pierce/Thermo, Bonn, Germany) and a Fusion SL camera system (Peqlab, Erlangen, Germany).

## Figures and Tables

**Table 1:** MD simulations conducted in this work. Each gray row corresponds to an MD set of simulated system types described in the flowchart (Figure 1). The blue and red rows specify the acyl chain lengths and protein form (WT/mutant), respectively. Other lines are a system composition with a number of entities rendered in subscript, and MD duration along with the number of independent replicas.

System Composition	MD Length (ns)	Number of Replicas
***Acyl-CoA in Lipid Bilayer***		
Acyl: C12/C14/C16/C18/C20		
Acyl-CoA_2_/POPC_176_/POPE_84_/POPI_28_/Water_25481_/Na^+^_88_/Cl^−^_50_	300	5
**C16**-CoA_2_/POPC_288_/Water_25481_/Na^+^_88_/Cl^−^_50_	300	1
***DHHC Enzyme in Lipid Bilayer***		
Protein: hDHHC20/hDHHC20^S29F^/hDHHC20^Y181A^ *(starting structure PDB ID: 6BMN)*		
Protein/POPC_144_/POPE_72_/POPI_25_/Water_24565_/Na^+^_72_/Zn^2+^_2_/Cl^−^_59_	1000	3
Protein/POPC_240_/Water_25004_/Na^+^_60_/Zn^2+^_2_/Cl^−^_72_	700–900	3
***DHHC Enzyme with Acyl-MEA in Lipid Bilayer***		
Protein: hDHHC20/hDHHC20^S29F^/hDHHC20^Y181A^ *(starting structure PDB ID: 6BML)*		
Acyl: C12/C14/C16/C18/C20		
Protein/Acyl-MEA/POPC_145_/POPE_69_/POPI_26_/Water_24558_/Na^+^_76_/Cl^−^_58_	500	15
***Acylated DHHC Enzyme in Lipid Bilayer***		
Protein: hDHHC20/hDHHC20^S29F^/hDHHC20^Y181A^ *(starting structure PDB ID: 6BML)*		
Acyl: C12/C14/C16/C18/C20		
Acyl-Protein/POPC_146_/POPE_71_/POPI_26_/Water_24555_/Na^+^_72_/Zn^2+^_2_/Cl^−^_58_	500	15
***DHHC Mutants (Predicted in Silico) with C18(16)-MEA in Lipid Bilayer***		
Mutant: V185G/S29A/V185A/S217A/L213A/S29A+V185A/V185G+S217A/A32L/Y33W/F58W/F62W/F184W/V185I/A186L/L213F *(starting structure PDB ID: 6BML)*		
Mutant/POPC_145_/POPE_69_/POPI_26_/Water_24570_/Na^+^_80_/Cl^−^_62_	100–500 *	15

*—details on these systems are presented in Table S1.

## Data Availability

The data presented in this study are freely available at the Zenodo archive (10.5281/zenodo.6497473): (1) the starting structures, topologies, and trajectories of the following systems: (2) free hDHHC20 enzyme and two of its mutants (S29F and Y181A) in the unbound state; (3) the pre-acylation step, when hDHHC20 accommodates MEA without a chemical bond formation; and (4) the post-autoacylation step, when hDHHC20 is covalently bound to the fatty acid residue.

## Supplementary Materials

The following supporting information can be downloaded at: https://www.mdpi.com/article/10.3390/ijms23095091/s1.
