# Supplementary material for: Molecular Dynamics of DHHC20 Acyltransferase Suggests Principles of Lipid and Protein Substrate Selectivity
Source: Int J Mol Sci. 2022 May 3;23(9):5091. doi: 10.3390/ijms23095091 (PMC9105814; doi:10.3390/ijms23095091)
Supplement: Supplementary file 1 [file ijms-23-05091-s001.zip › ijms-1682506-supplementary.pdf]

Supplementary information

Molecular dynamics of DHHC20 acyltransferase suggest principles of lipid and protein substrate selectivity

Irina S. Panina[1,2], Nikolay A. Kyrlov[1,2], Mohamed Rasheed Gadalla[3], Elena T. Aliper[1], Larisa V. Kordyukova[4], Michael Veit[3], Anton O. Chugunov[1,2,5,*] Roman G. Efremov[1,2,5]

[1]Shemyakin-Ovchinnikov Institute of Bioorganic Chemistry, Russian Academy of Sciences, Moscow, Russia

[2]National Research University Higher School of Economics, International Laboratory for Supercomputer Atomistic Modelling and Multi-Scale Analysis, Moscow, Russia

[3]Institute of Virology, Department of Veterinary Medicine, Free University Berlin, Berlin, Germany

[4]Belozersky Institute of Physico-Chemical Biology, Lomonosov Moscow State University, Moscow, Russia

[5]Moscow Institute of Physics and Technology (State University), Dolgoprudny, Moscow Region, Russia

*Corresponding author. Email: batch2k@yandex.ru

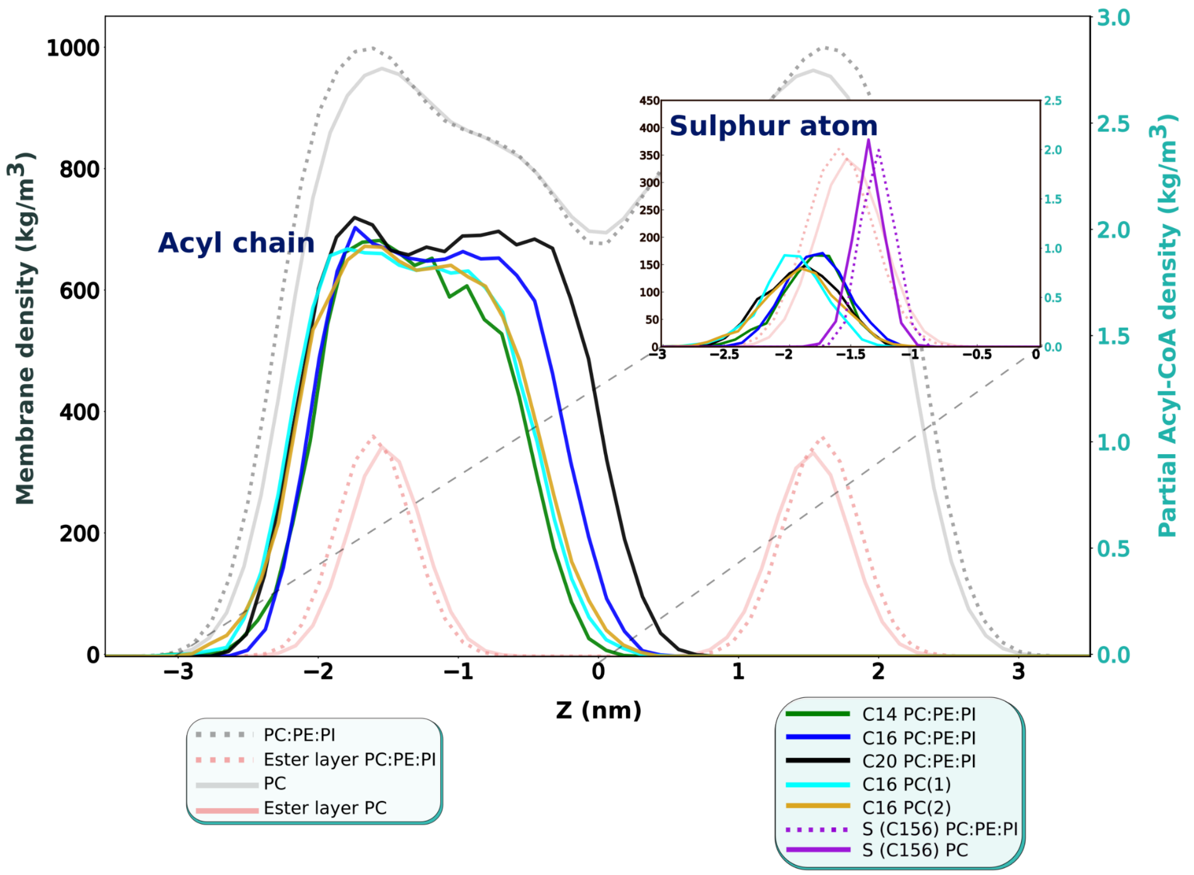

Supplementary Figure S1. Interfacial position of acyl-CoA's sulfur atom is independent of the acyl chain's length and matches the hDHHC20's Cys^{156} sulfur atom. Shown in the plot are MD-averaged density profiles of the membrane-embedded acyl-CoA (*right axis*) and the membrane lipids (*left axis*) as a function of the distance from the bilayer center (Z). The main plot shows total membrane lipid density (*gray*) and that of the ester layers (*coral*) in a POPC bilayer (*solid lines*) and a POPC:POPE:POPI mixture (*dotted lines*); and acyl-CoAs' acyl chain densities, colored as specified in the legend. Note the ladder-like positioning of the right sides of the distribution curves, echoing the chain length. *The inset* shows densities of the sulfur atoms of the same acyl-CoAs and Cys^{156} of the hDHHC20 (*solid and dotted purple lines* correspond to this parameter in a POPC-based system in a POPC:POPE:POPI mixture, respectively). Note that: 1) distributions don't depend on the acyl chain's length; 2) distributions for acyl-CoA and hDHHC20 partially overlap, allowing optimal positioning and DHHC's autoacylation. Also note that in these simulations two acyl-CoA molecules were initially placed into solvent and subsequently none, one or two of them embedded themselves into the membrane (see *Methods* for details). Thus, two distributions for C16 PC (*cyan* and *orange*) mean that both molecules are embedded in the bilayer. Density profiles were only calculated upon complete accommodation.

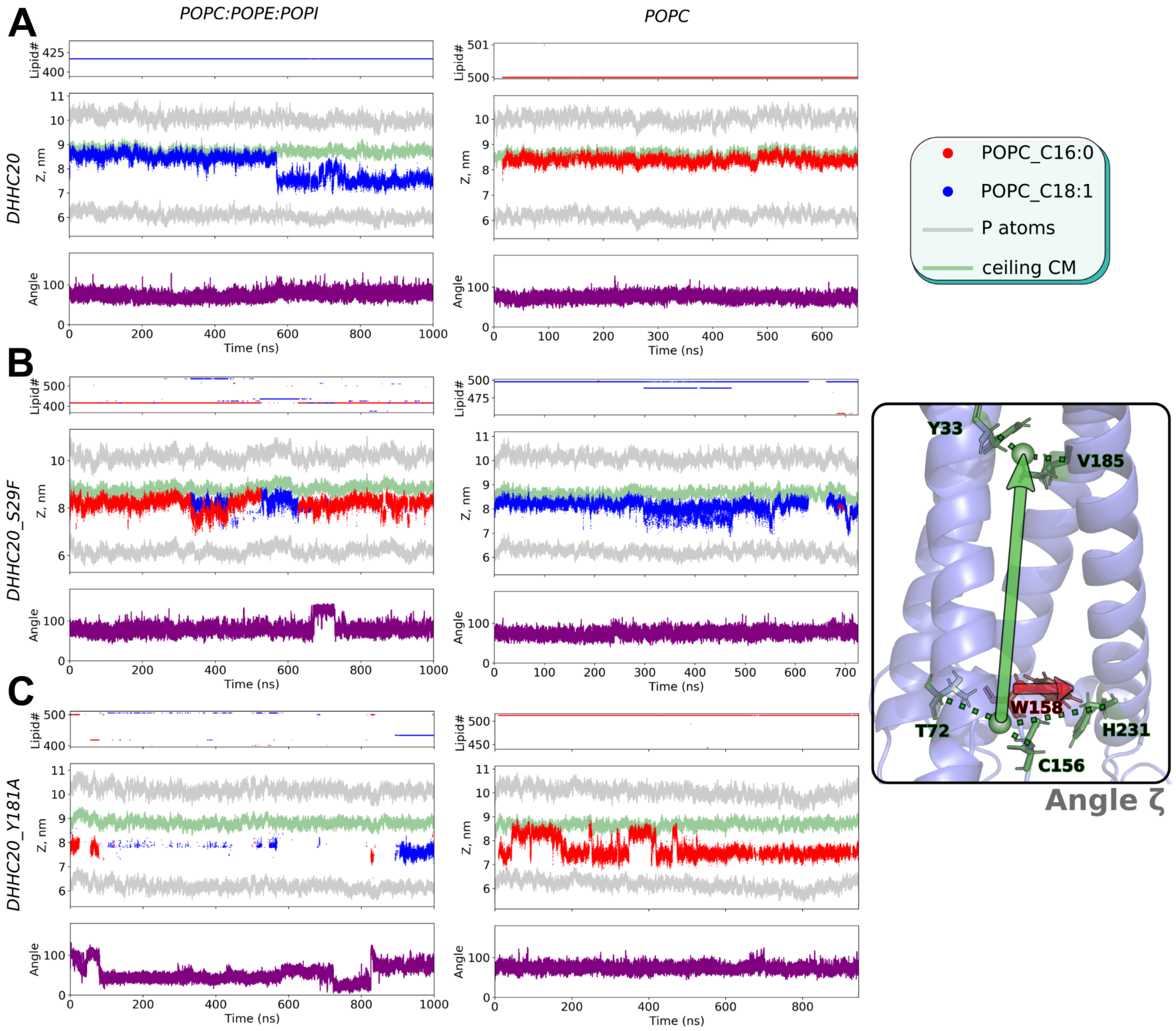

Supplementary Figure S2. Membrane lipid accommodation in the hDHHC20 cavity. Each panel demonstrates the time-dependent lipid tail deployment, depth of insertion and position of Trp^{158} for hDHHC20 (**A**), $hDHHC20^{S29F}$ (**B**), and $hDHHC20^{Y181A}$ (**C**) in a phospholipid mixture bilayer (*left*) and in a pure POPC bilayer (*right*). *The upper plot* specifies the trajectory ID and the chain of the phospholipid molecule inside the cavity; *red* and *blue* graphs represent saturated and unsaturated chains, respectively. *The middle graph* shows the depth of penetration of the tail tip inside the protein cavity, measured as the Z-distance between the lipid tip (*red* and *blue* graphs represent saturated and unsaturated chains, respectively) and the ceiling's center of mass (CM, rendered in *light green*). *The lower plot* illustrates the evolution of the ζ angle between Trp^{158} plane and the cavity axis (see inset) in the course of MD simulations.

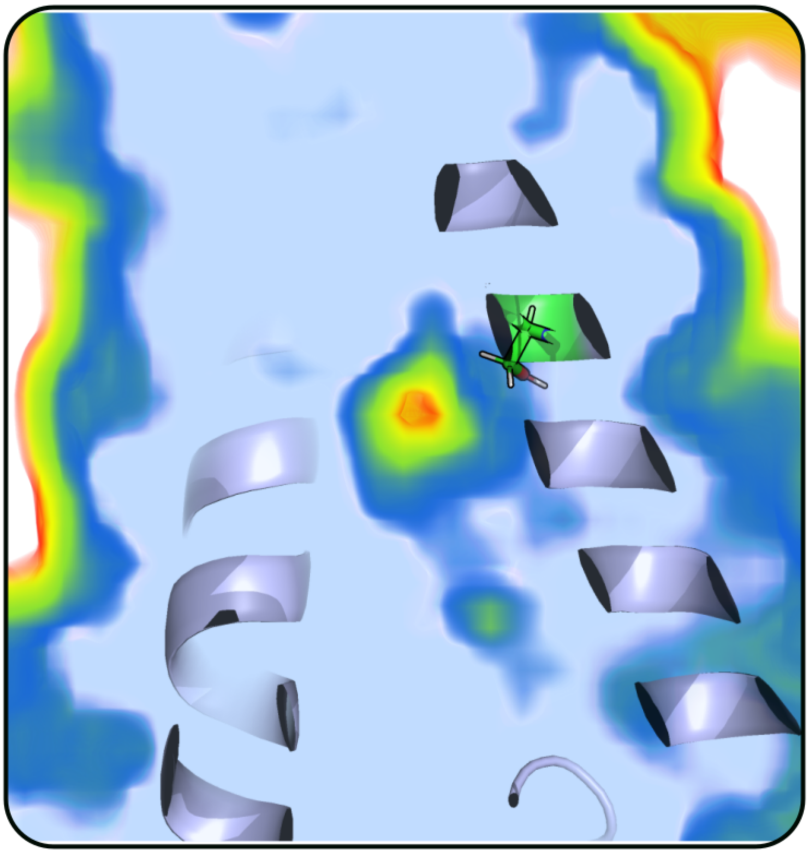

Supplementary Figure S3. Empty protein cavity inside hDHHC20^{Y181A}, which is the only example of a long-lasting absence of a lipid tail inside (see Fig. S2C, *left*). In this case the "entrance gate" of Trp^{158} side chain remained closed (see Fig. 2, *inset*). The cavity is not empty, instead characterized by the decreased protein density; only right under the "ceiling" can some empty space be found.

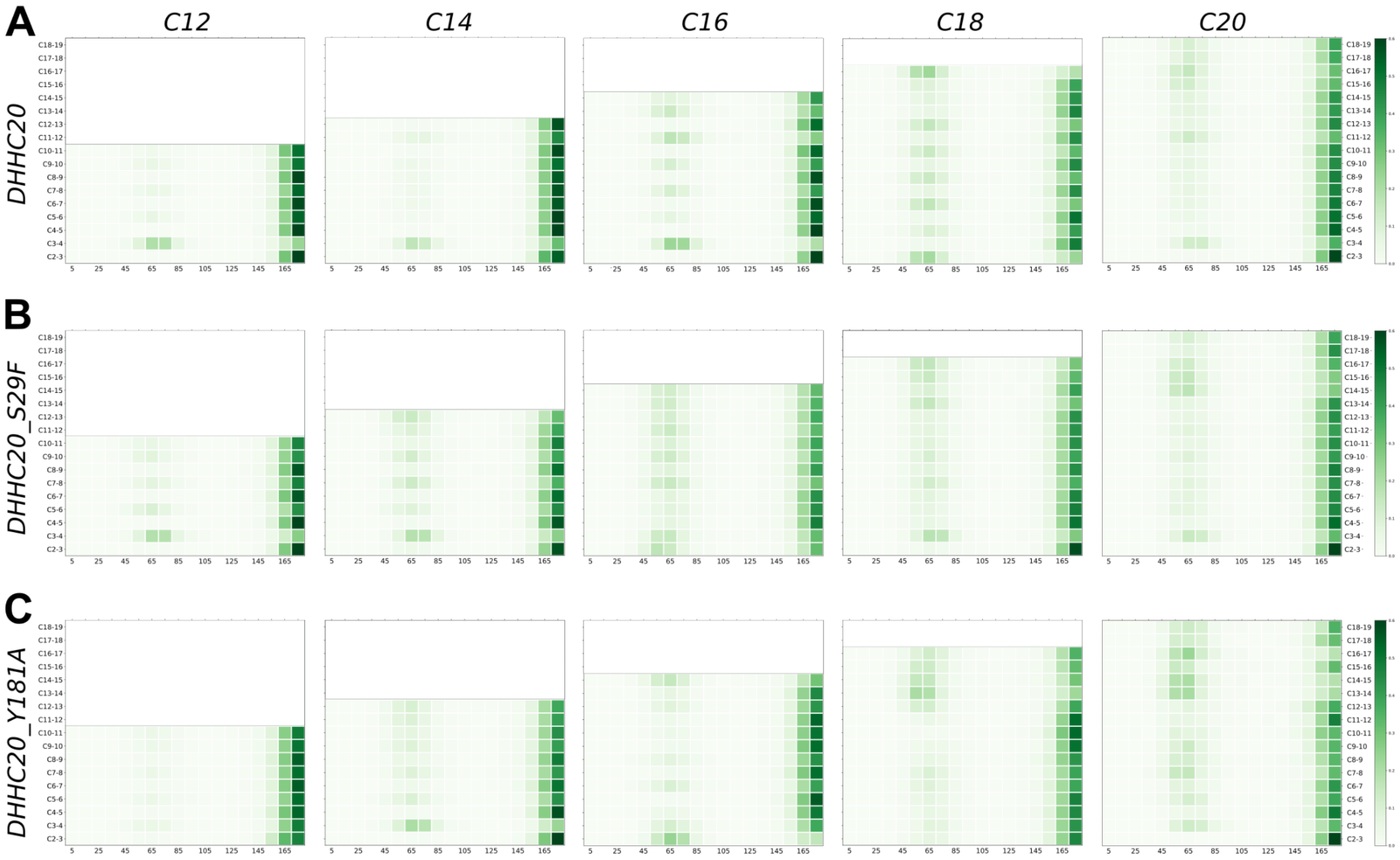

Supplementary Figure S4. MD-averaged probability density of the acyl chain's dihedral angles inside the acylated DHHC protein variants. Each plot presents data for a separate MD trajectory for hDHHC20^{WT}, hDHHC20^{S29F} or hDHHC20^{Y181A}, acylated with substrates C12 to C20. While the Y coordinate shows a certain dihedral, X is its value. The more intense the color is, the more populated is the state: the most frequently occurring are ≈180° angles (all-trans state), and there is an additional tense (≈65°) state.

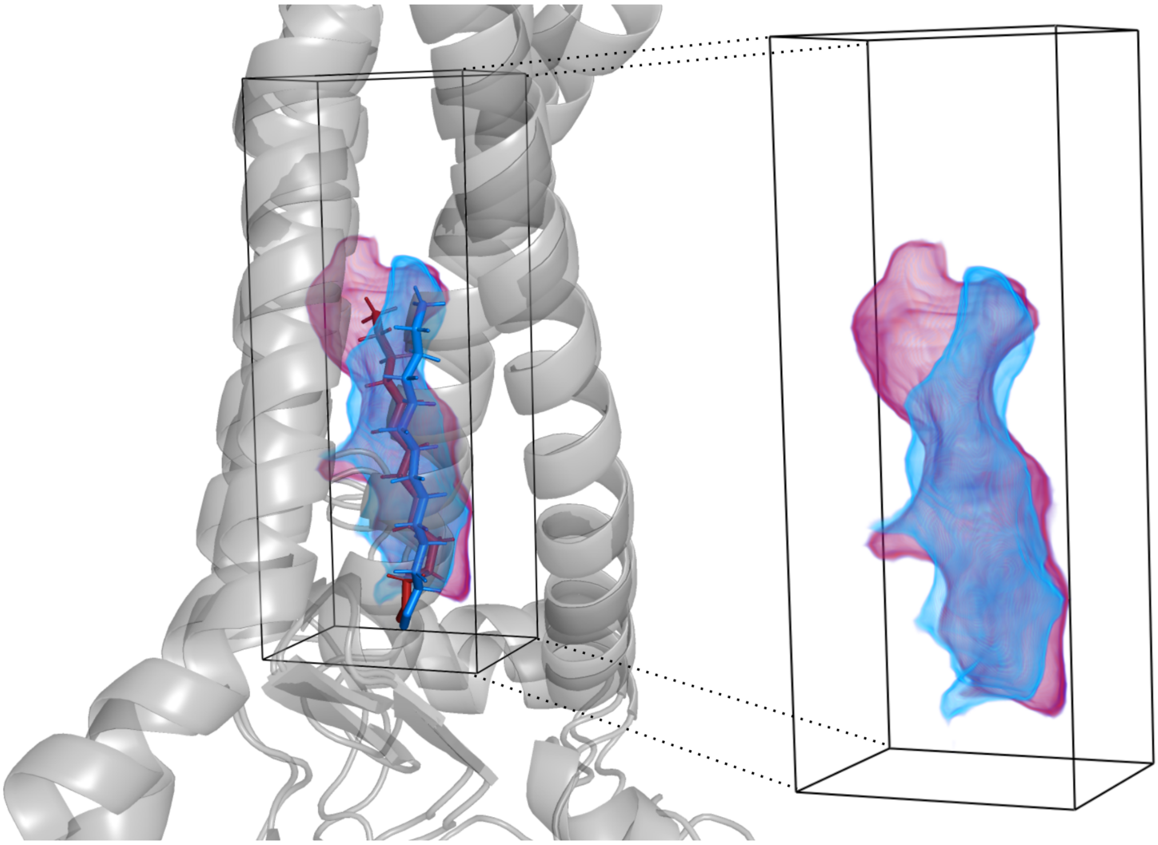

Supplementary Figure S5. Free cavity volume of acylated hDHHC20 and its Y181A mutant. Aligned structures of myristoyl-hDHHC20 (*blue*) and myristoyl-hDHHC20^{Y181A} (*red*) from MD calculations. The protein's backbone structures are shown as a *gray cartoon*. Semi-transparent *blue* and *red* volumes contour low-density areas in hDHHC20 and hDHHC20^{Y181A}, respectively. The volumes are averaged over 100 ns.
Note the equivalent cavity length and remarkable additional volume on the side in the hDHHC20^{Y181A} mutant.

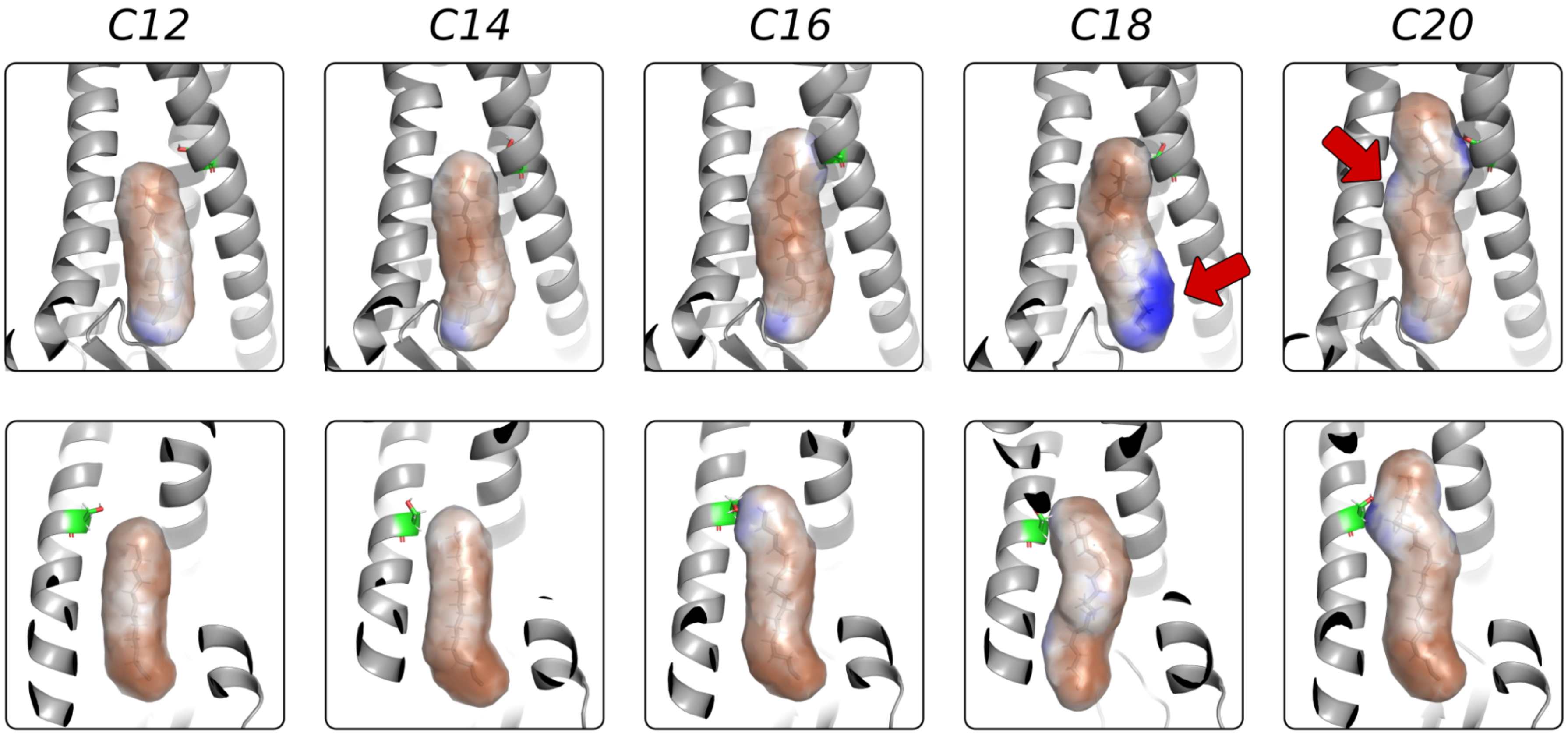

Supplementary Figure S6. Hydrophobic (mis)match for acyl chains (C12..C20) inserted into hDHHC20 protein. Each pair of panels (vertically stacked) show the hydrophobicity of the acyl chain's environment (protein + membrane), calculated in accordance with the Molecular Hydrophobicity Potential (MHP) formalism. The *upper* panel shows the view from the "entrance", while the *lower panel* renders the same objects rotated 180° around the vertical axis. The hydrophobic and hydrophilic environments are shown in *brown* and *blue*, respectively. The MHP of the acyl chains MHP is not shown, since they are completely hydrophobic. Ser^{217} (rendered as *green sticks*) marks the "ceiling" level. Note that C12 and C14 perfectly match the cavity in terms of MHP, while C16 already has a slight hydrophilic spot casted by Ser^{217}. Longer chains exhibit a higher level of the mismatching: C18 twists and bulges out of the protein (*red arrow*), contacting the lipid milieu (in this case the blue spot only indicates the molecule's exposure); C20 has two mismatched spots: Ser^{217} and Ser^{29} (*red arrow*).

Table S1. Assessment of hDHHC20 *in silico* mutants for their potential to accommodate a C18-acyl substrate. Each table line is an extended version of the last section from Table 1. The positioning of the C18 chain tip within the cavity of calculated DHHC mutants in terms of parameter *D* and *ΔD* values (see Fig. 6A for definition) is presented. The mutants with noticeably positive *ΔD* values are highlighted in green as possibly selective for C18-acyl.

Mutant	*D*	*ΔD**	MD length (ns)	Comment
WT	−1.7 ± 0.7	-	500	*The population of the major state is 76%. There are also two minor states:*<br>• *D* = 6.7 ± 0.6 (12%)**<br>• *D* = −12.7 ± 0.7 (1%)***
V185G	0.9 ± 0.7	2.6	500	*The population of the major state is 89%. There is another state:*<br>*D* = 3.4 ± 0.5 (7%)
S29A	−1.7 ± 0.9	0	500	*Calculation is performed with palmitate (C16) as substrate*
V185A	−1.9 ± 0.8	−0.2	500	*Palmitate (C16) is embedded*<br>*The population of the major state is 89%. There is another state:*<br>*D* = −4.9 ± 1.1 (7%)
S217A	−1.3 ± 0.8	0.4	500	
L213A	−2.7 ± 0.7	−1	280	*Calculation is performed with palmitate (C16) as substrate*
S29A+V185A	3 ± 0.8 (1)<br>−2 ± 0.7 (2)	4.7<br>−0.3	500	*The only system with two equally frequent states: 54% (1) and 43% (2)*
V185G+S217A	1.6 ± 0.5	3.3	500	
A32L	−2 ± 0.6	−0.3	315	
Y33W	−1.6 ± 0.9	0.1	500	
F58W	−2 ± 0.7	−0.3	100	
F62W	−1.5 ± 0.7	0.2	100	
F184W	−1.9 ± 0.7	−0.2	120	
V185I	−1.9 ± 0.9	−0.2	250	
A186L	−1.8 ± 0.6	−0.1	330	
L213F	−1.9 ± 0.6	−0.2	170	*The population of the major state is 87%. There is another state:*<br>*D* = −6.4 ± 1 (10%)

* — a negative value means that the tip of C18 is lower than in WT, a positive value indicates that the “ceiling” is moved up, and the cavity probably may accommodate aC18-acyl substrate.
** — a rare event of arbitrary rising of the ceiling and, accordingly, acyl tail raising.
*** — this pronounced negative value corresponds to the temporary acyl chain exit from the cavity and subsequent re-entrance (see Fig. 6B).
